# Behavioral Deficits in Mice with Postnatal Disruption of *Ndel1* in Forebrain Excitatory Neurons: Implications for Epilepsy and Neuropsychiatric Disorders

**DOI:** 10.1093/texcom/tgaa096

**Published:** 2021-01-07

**Authors:** Cezar Gavrilovici, Yulan Jiang, Ivana Kiroski, Toni-Lee Sterley, Milene Vandal, Jaideep Bains, Sang Ki Park, Jong M Rho, G Campbell Teskey, Minh Dang Nguyen

**Affiliations:** 1 Departments of Neurosciences & Pediatrics, University of California San Diego, Rady Children’s Hospital San Diego, San Diego, CA 92123, USA; 2 Departments of Clinical Neurosciences, Cell Biology and Anatomy, and Biochemistry and Molecular Biology, Hotchkiss Brain Institute, Calgary, AB T2N 4N1, Canada; 3 Departments of Physiology and Pharmacology, University of Calgary, Calgary, AB T2N 4N1, Canada; 4 Department of Life Sciences, Pohang University of Science and Technology, Pohang 37673, Korea; 5 Department of Cell Biology and Anatomy, Hotchkiss Brain Institute, Calgary, AB T2N 4N1, Canada

**Keywords:** epilepsy, hippocampus, Lis1 Reelin, mouse behavior, Ndel1, Nde1, schizophrenia

## Abstract

Dysfunction of nuclear distribution element-like 1 (Ndel1) is associated with schizophrenia, a neuropsychiatric disorder characterized by cognitive impairment and with seizures as comorbidity. The levels of Ndel1 are also altered in human and models with epilepsy, a chronic condition whose hallmark feature is the occurrence of spontaneous recurrent seizures and is typically associated with comorbid conditions including learning and memory deficits, anxiety, and depression. In this study, we analyzed the behaviors of mice postnatally deficient for Ndel1 in forebrain excitatory neurons (Ndel1 CKO) that exhibit spatial learning and memory deficits, seizures, and shortened lifespan. Ndel1 CKO mice underperformed in species-specific tasks, that is, the nest building, open field, Y maze, forced swim, and dry cylinder tasks. We surveyed the expression and/or activity of a dozen molecules related to Ndel1 functions and found changes that may contribute to the abnormal behaviors. Finally, we tested the impact of Reelin glycoprotein that shows protective effects in the hippocampus of Ndel1 CKO, on the performance of the mutant animals in the nest building task. Our study highlights the importance of Ndel1 in the manifestation of species-specific animal behaviors that may be relevant to our understanding of the clinical conditions shared between neuropsychiatric disorders and epilepsy.

## Introduction

Nuclear distribution element-like 1 (Ndel1) was initially characterized as a binding partner of the Lis1/Dynein motor protein complex that regulates microtubule (MT) organization and intracellular transport (Niethammer et al. 2000; Sasaki et al. 2000; Chansard, Hong, et al. 2011a; Chansard, Wang, et al. 2011b). The protein is now considered an important integrator of the cytoskeleton via regulation of MTs, actin-associated proteins and intermediate filaments, and an effector of cell signaling mediated by kinases and GTPases (Chansard, Hong, et al. 2011a; Chansard, Wang, et al. 2011b). During brain development, Ndel1 is implicated in several fundamental processes including proliferation and differentiation of neuronal precursors (Liang et al. 2007; Stehman et al. 2007; Ye et al. 2017), neuronal migration (Shu et al. 2004; Sasaki et al. 2005; Youn et al. 2009; Takitoh et al. 2012; Okamoto et al. 2015), morphogenesis and maturation (Kamiya et al. 2006; Shim et al. 2008; Youn et al. 2009; Hayashi et al. 2010; Jiang et al. 2016; Kuijpers et al. 2016; Saito et al. 2017; Woo et al. 2019). In the adult brain, Ndel1 maintains the positioning and functioning of CA1 principal neurons in dorsal hippocampus (Jiang et al. 2016). Specifically, mice with a targeted postnatal disruption of the Ndel1 gene in forebrain excitatory neurons (Ndel1 CKO) exhibit fragmentation of MT structure in CA1 pyramidal neurons that cause dendritic and synaptic pathologies and render them hyperexcitable (Jiang et al. 2016). Further, Ndel1-deficient CA1 pyramidal neurons undergo postnatal dispersion, independently of neuronal migration defects. The phenotype arises well after the formation of the CA1 and is exacerbated in adulthood as the mice age (Jiang et al. 2016). These cellular, molecular, and anatomical abnormalities in the CA1 are likely responsible for the spatial learning and memory deficits, and self-generated seizure activity observed in the mutant mice (Jiang et al. 2016; Kiroski et al. 2020). Remarkably, the dendritic/synaptic pathologies, neuronal dispersion, and cognitive dysfunction can be mitigated upon replenishment of the glycoprotein Reelin in the hippocampus of Ndel1 CKO (Jiang et al. 2016; Kiroski et al. 2020). The longer MTs, improved dendritic arborization and ameliorated electrophysiology in the Ndel1 CKO mice treated with Reelin (Jiang et al. 2016) are associated with a normalization of the excessive levels of Lis1 (Kiroski et al. 2020). Since increased dosage of Lis1 causes MTs fragmentation (Smith et al. 2000), we reasoned that Reelin confers neuroprotection in the mutant mice via Lis1-dependent MTs stabilization. In sum, these results provide evidence that Ndel1 and Reelin co-operate to maintain CA1 function during postnatal life.

Postmortem and human genetic studies implicate Ndel1 in schizophrenia, a neuropsychiatric disease characterized by memory problems, hallucinations, delusions, anxiety, depression, and social withdrawal (Lipska et al. 2006; Burdick et al. 2008; Nicodemus et al. 2010; Gadelha et al. 2016; Bradshaw and Hayashi 2017). The levels of Ndel1 are lower in postmortem tissues of people with schizophrenia when compared with control tissues (Lipska et al. 2006), whereas they appear to be upregulated in the first episode of psychosis (Ota et al. 2019). Interestingly, postmortem brain analysis revealed decreased expression of Ndel1 in resected human epileptic sclerotic tissues (but not in nonsclerotic specimens) (Gavrilovici et al. 2020). Experiments using cell cultures and animal models (Wu et al. 2014; Choi et al. 2016; Jiang et al. 2016; Kiroski et al. 2020; Zhu et al. 2020) also point to a role for Ndel1 in seizure activity. Moreover, multidisciplinary (i.e., epidemiological, clinical, neuropathological, and neuroimaging) studies provide evidence for similarities between schizophrenia and epilepsy, and particularly temporal lobe epilepsy (TLE) when considering the common pathophysiology in the hippocampus (Cascella et al. 2009; Kandratavicius et al. 2014; Wang et al. 2017; Nakahara et al. 2018). Indeed, some individuals with epilepsy are prone to develop psychotic symptoms consistent with those seen with schizophrenia. Conversely, people with schizophrenia are predisposed to develop seizures while patients with both diseases die much earlier (Andersen et al. 2019). Taken together, these results provide evidence that Ndel1 dysfunction may be important for the pathogenic mechanisms and manifestation of symptoms and/or comorbidities common to both epilepsy and schizophrenia.

Like Ndel1, the glycoprotein Reelin plays an important role during neuronal migration (Sekine et al. 2014; Chai and Frotscher 2016). Secreted by interneurons in the postnatal brain, Reelin also specifies CA1 pyramidal cell maturation (Kupferman et al. 2014). In the adult brain, Reelin regulates neuronal plasticity (Beffert et al. 2005; Qiu et al. 2006; Qiu and Weeber 2007), increases neurotransmission (Bal et al. 2013), and enhances long-term potentiation in hippocampal slice cultures (Weeber et al. 2002). Mutations in *reelin* that reduce the protein levels account for familial forms of TLE, the most common seizure disorder in adults (Heinrich et al. 2006; Dazzo et al. 2015). The levels of Reelin are also significantly lower in people diagnosed with schizophrenia and psychotic bipolar disorders (Fatemi 2001; Ishii et al. 2016).

In this study, we further characterized the role for Ndel1 in mouse behaviors that may be relevant to clinical manifestations shared between schizophrenia and epilepsy. We analyzed the behaviors of Ndel1 CKO mice in species-specific tasks and surveyed the expression of signaling molecules that may linked to these behaviors. Finally, we tested the effects of Reelin on the performance of the mutant mice in the nesting behavioral task.

## Methods

### Generation of Ndel1 CKO Mice

**Figure 1:**
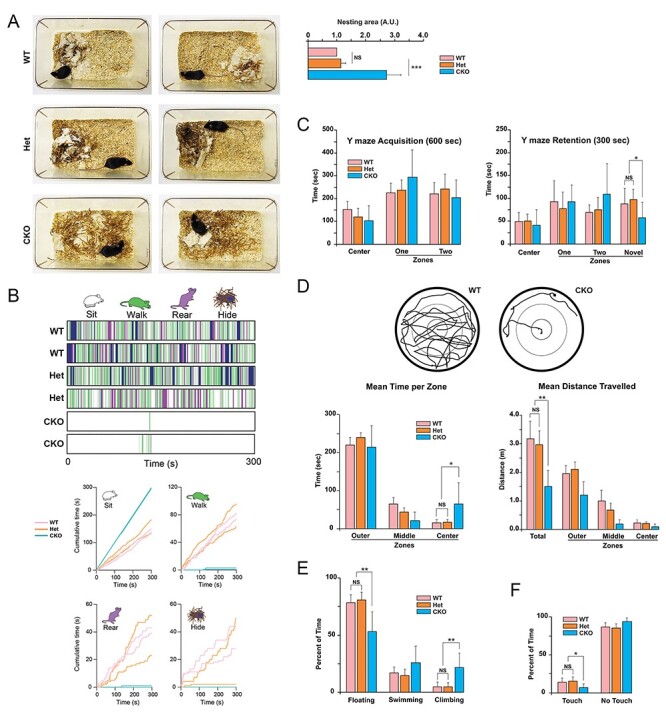
Behaviors of Ndel1 CKO mice in species-specific tasks. (*A*) Nest building: Ndel1 CKO mice, heterozygous CKO, and WT littermates were monitored for their ability to build nest around 5–8 weeks of age, after cage change. Representative photographs of nests built by mice with the respective genotypes 24–48 h after cage change. The Ndel1 CKO failed to build their nest. This phenotype was highly penetrant and observed in more than 95% of the Ndel1 CKO analyzed. A total of 120 mice were monitored for this phenotype over a period of ~5 years. The bar graph depicts the relative area with bedding material in the cages of WT, Het and CKO mice. Mean ± SD, ^*^^*^^*^P<0.005, NS: nonsignificant. *N* = 3–8 mice, average age: ~7 weeks. (*B*) In-cage behavioral ethograms for the 2 WT, 2 Het, and 2 CKO shown in (*A*) and Supplementary Videos 1–6. The ethograms illustrate the 4 mutually exclusive behaviors (sitting, walking, rearing, hiding under nesting material) displayed by the mice at each time point over 300 s. The graphs show the cumulative time each mouse engaged in 1 of the 4 behaviors. (*C*) Y maze: During the 600 s acquisition of the Y maze mice of all three genotypes (WT, Het, and CKO) spent equivalent amounts of time in zones one and two. During the 300 s retention trial Ndel1 CKO mice spent significantly less time in the novel zone. Mean ± SD, ^*^P<0.05. (*D*) Open Field: Representative examples of paths taken by a WT and a CKO mouse during 300 s in the open field and quantification of the total time spent as well as the time spent in the outer, middle, and center zones. Ndel1 CKO mice spent significantly longer in the center zone compared to the other two genotypes. Quantification of the total distance traveled as well as the distance traveled in the outer, middle and center zones. Ndel1 CKO mice traveled significantly less distance than WT and Het mice. Mean ± SD, ^*^P < 0.05, ^*^^*^P < 0.01. (*E*) Forced swim: Ndel1 CKO mice spent significantly more time climbing and significantly less time floating than the other two genotypes (WT and Het). Mean ± SD, ^*^^*^P < 0.01. (*F*) Dry cylinder: Ndel1 CKO mice spent less time touching the walls (more time not touching the walls) compared with the other two genotypes. Mean ± SD, ^*^P < 0.05. 7 mice of each genotype were subjected to the tests in (*B*-*E*) at 7 weeks of age.

**Figure 2:**
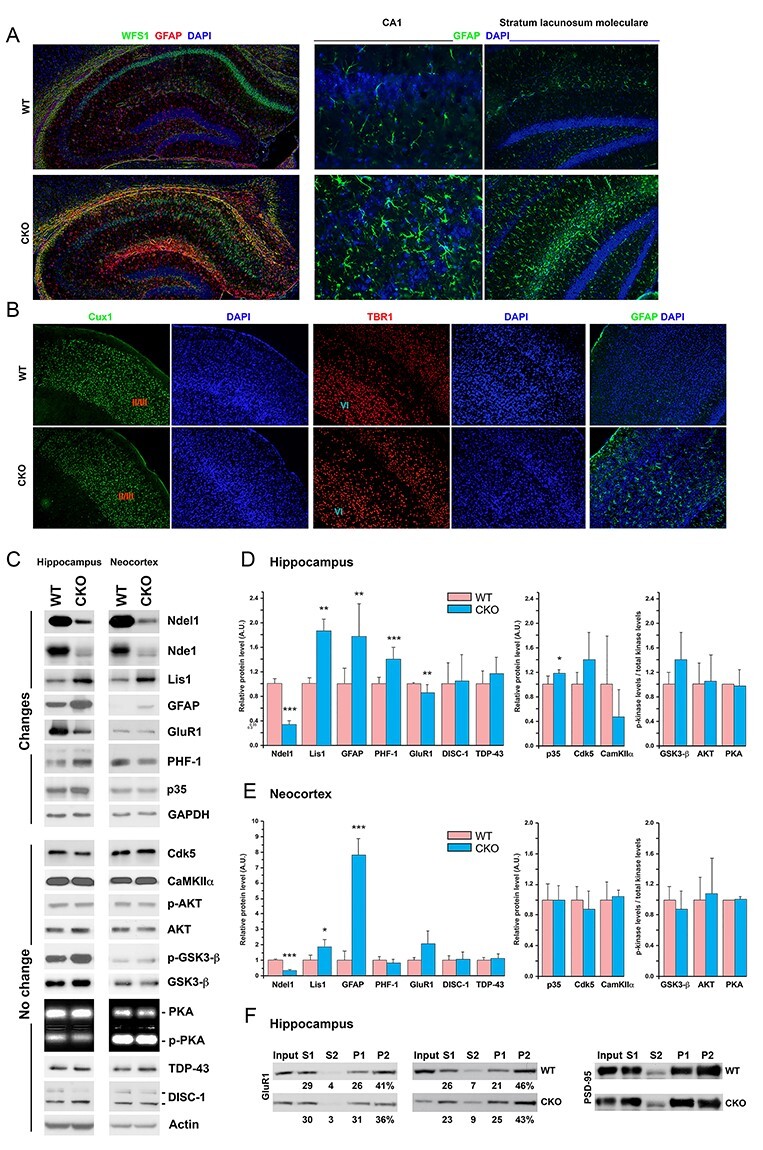
Gross anatomy and molecular changes in the hippocampus and neocortex of Ndel1 CKO mice. (*A*) Bilayered CA1 of Ndel1 CKO mice at ~7 weeks of age is associated with intense astrogliosis in the CA1 and stratum lacunosum moleculare. GFAP detects astrocytes while WFS1 labels preferentially CA1 pyramidal neurons. (*B*) Neocortical lamination of Ndel1 CKO mice appears intact at 12 weeks of age (the average lifespan of the mutant mice is 10 ± 4 weeks, see Jiang et al. 2016; Kiroski et al. 2020), as indicated by immunofluorescent staining with Cux1 (that label neurons of layers 2 and 3) and TBR1 antibodies (that label neurons of layer 6). (*C*) Levels of proteins potentially linked to Ndel1 biology, Ndel1 CKO phenotypes, epilepsy and/or schizophrenia in the neocortex and hippocampus of Ndel1 CKO mice. (*D*) Bar graph quantifications for the levels of proteins (in arbitrary units - A.U.) and ratios of phospho-kinase/total kinase illustrated in (*C*) in the hippocampus, *n* = 3–8 mice. (E) Bar graph quantifications for the levels of proteins (in arbitrary units - A.U.) and ratios of phospho-kinase/total kinase illustrated in (*C*) in the neocortex, *n* = 3–8 mice. For (*D*-*E*): mean ± SD; Student’s *t*-test; ^*^P < 0.05, ^*^^*^P < 0.01, ^*^^*^^*^P < 0.001; *n* = 3–8 mice, average age: ~9 weeks. (*F*) Crude synaptosome fractionation determines the amounts of GluR1 found in the preclear lysate (S1), cytosolic fraction (S2), pellet 1 (P1), or crude synaptic compartment (P2). The amount of protein in each fraction is estimated in percentage. Representative blots from hippocampal lysates of 2 Ndel1 CKO and 2 WT out of 6 animals of each genotype depict a subtle decrease of GluR1 (3.2 ± 3.1%; mean ± SD, *n* = 6; Student’s *t*-test; *P* = 0.0468) in the synaptic compartment. PSD-95, a postsynaptic marker, was used a control for the procedure. Average age of the animals: 9.5 weeks.

**Figure 3:**
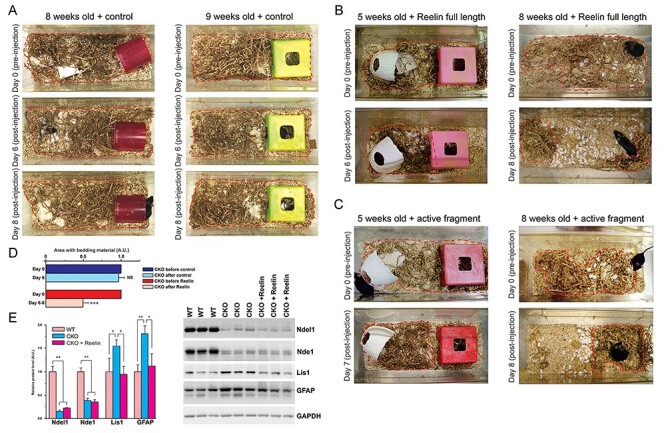
Reelin improves the performance of the Ndel1 CKO mice in the nest building task. (*A*-*C*) Representative photographs depicting the performance of Ndel1 CKO mice injected at 5, 8, or 9 weeks of age with (*A*) the purified full length protein (*n* = 3), (*B*) a commercially available central active fragment of Reelin (*n* = 2) or (*C*) a control solution (*n* = 3). All mice were monitored for their performance after injection for up to 8 days. Red traces delimit the area with bedding material. (*D*) Bar graph showing the relative area with bedding material in the cage before and after control or Reelin injection in Ndel1 CKO mice. Mean ± SD, Student’s *t*-test, ^*^^*^^*^P < 0.001, NS: nonsignificant; *n* = 8 mice in total, average age: ~8 weeks; A.U.: arbitrary units. (*E*) Levels of Ndel1, Lis1, and GFAP in WT, Ndel1 CKO mice, and Ndel1 CKO mice injected with Reelin. Data were analyzed and compared using One-way ANOVA and Tukey’s multiple comparison test. ^*^P < 0.05, ^^*^^*^^P < 0.01; *n* = 3/group.

Forebrain excitatory neuron-specific knockout mice for Ndel1 were created by breeding CaMKIIα-Cre transgenic mice (Jackson laboratory, Tsien et al. 1996) with Ndel1-LoxP mice generated previously (Sasaki et al. 2005). These mice with the Cre element and both Ndel1 alleles deleted are designated Ndel1 conditional knockout (CKO; –/–) at ~1 month of age when the Ndel1 protein is knocked down (Jiang et al. 2016). All mice were genotyped by PCR prior to experimentation. Wild type (+/+, *n* = 7), heterozygous Ndel1 CKO (+/–, *n* = 7), and homozygous Ndel1 CKO (–/–, *n* = 7) mice of both sexes (9F, 12M) were used in the behavioral experiments in Figure 1*C*-*F*, the gender distribution as per genotype is the following: WT (4F +3M), Het (3F + 4M), and CKO (2F + 5M). Figure 1*A*, quantifications of the nesting area were performed on 5 WT (4F, 1M), 4 Het (2F, 2M), 11 CKO (4F, 7M). For the in-cage behavioral ethograms in Figures 1*B* and 2, WT (2F), 2 Het (1F, 1M), and 2 CKO (2M) were used. For Figure 3, the nesting area of 8 CKO (3F, 5M) prior and after injection was used for quantifications. 5 mutant mice (2F, 3M) were injected with Reelin: 3 received the full-length protein and 2 received the central fragment purchased commercially. 3 CKO (1F, 2M) were injected with the control solution. The colony room was kept at 21°C under a 12:12 light–dark cycle (lights on at 08:00). Mice received food and water ad libitum. The mice were housed and handled according to Canadian Council on Animal Care guidelines and experimentation approved by the Health Sciences Animal Care Committee.

### Behavioral Tasks

#### In-Cage Behaviors

Six mice (2 WT, 2 Het, 2 CKO) were video recorded in their home cage using an overhead camera for 5 min. Spontaneous home cage behaviors were manually annotated (github.com/tsterley) by an experimenter blind to the genotype of the mice. Mutually exclusive behaviors were scored as in Füzesi et al. (2016), specifically “sit” (the mouse is stationary with the exception of surveying or air-sampling), “walk” (the mouse changes location or turns, so long as front paws move), and “rear” (the mouse lifts front paws off the ground into the air or against the cage wall). When the mouse was obscured by nesting material the behavior was annotated as “hide.” The behaviors annotated are typical behaviors exhibited by mice when they are alone in their home cages (Füzesi et al. 2016). Walking and rearing are typically viewed as exploratory behaviors.

#### Nest Building

Mice aged between 5 and 10 weeks old (*n* = 120) were monitored for their ability to build nest from bedding material during cage changes. At 7 weeks of age, mice underwent a battery of behavioral testing on day 1: nesting building, Y-maze in the am and open field in the pm. The nesting area (expressed in arbitrary units) was calculated by delimiting the area with bedding material 24–72 h after moving single animals to new cages. On day 6 (following the water maze task, published in Kiroski et al. [2020], Fig. 1), the mice were subjected in the pm to forced swim and the next day in the am, to the second forced swim, and in the afternoon to the wall touch (Schallert cylinder) task.

#### Y-Maze

The maze was constructed from three Plexiglas zones of equal size joined together in a *Y* configuration measuring 40 cm in length, 10 cm in width, and 12 cm in height. The task consisted of two trials: an acquisition trial followed by a retention trial. During the acquisition trial each mouse individually explored two of the three arms for a duration of 600 s while the third arm was blocked. Mice were then returned to their home cages for 30 min prior to a 300 s retention trial which consisted of all three zones available. Mice were individually tested, and Windex was used to clean the apparatus between trials. All sessions were video captured with an image tracking system (Harvard Canada, Saint-Laurent, Quebec, CAN) and the total time spent in each arm was determined.

#### Open Field Task

The Open Field task was conducted on a white, circular wooden table, 155 cm in diameter, elevated 64 cm above the floor. The same image tracking system used for the Y-maze was also used for this task. Mice were individually placed in the center and allowed to freely move about the apparatus for 300 s while being video recorded.

#### Forced Swim and Dry Cylinder Tasks

The Forced Swim task involved placing the mice individually into a 2 L glass beaker filled with warm (24.5 + 0.5°C) water. The first exposure lasted 15 min followed by a 24-h break. The second exposure lasted 300 s in which the time spent floating (immobile), swimming, and climbing the side of the container was recorded. Swimming was defined as both forepaws and hindlimbs paddling below water level, not against the side of the container, with the body approximately parallel to the surface of the water. This was in contrast to climbing, defined as forepaws paddling above water level, against the side of the container, with the body approximately vertical in the water or parallel to the side of the container. The Dry Cylinder task was conducted in the same 2 L glass beaker to examine exploratory tendencies and locomotor asymmetry. Mice were placed individually for 300 s in to same 2 L glass beaker, but it was empty. The total amount of time spent touching versus not touching the sides of the container with either forelimb was measured.

### Western Blot

Total protein extracts of dissected mouse hippocampi were obtained by homogenization in Triton X-100 (10 mM Tris-HCl, pH 7.5, 150 mM NaCl, 1 mM EDTA, pH 8.0, and 1% Triton X-100) buffer with cocktails of protease and kinase inhibitors. The protein concentration was estimated by the Bradford or DC assay (Bio-Rad Laboratories). Proteins were fractionated by SDS-PAGE and blotted on a nitrocellulose or PVDF membrane for western blot analysis. Membranes were incubated with specific antibodies for Ndel1 (homemade, see Niethammer et al. 2000; Smith et al. 2000; Nguyen et al. 2004), Nde1, Lis1, GFAP, TDP-43, DISC1, PKA, Cdk5, Akt, phospho-Akt, GSK3β, phospho-GSK3β, CaMKII, GluR1—see Table 1 for catalog numbers—PSD-95 (Neuromab 75-028), GAPDH (Abcam, Ab9482 HRP-conjugated), and actin (Chemicon, MAB1501, clone C4). The western blots were examined using a chemiluminescence kit from NEN Life Science. Quantifications were corrected with levels of housekeeping proteins, such as actin and GAPDH, and performed with the Labscan program (Image Master, 2D software version 3.10, GE Healthcare Pharmacia Biotech).

### Crude Synaptosomes Preparation

Mouse brain was homogenized in 7 ml of 10 mM HEPES, 0.32 M sucrose at 1000 g at 4°C for 10 min. Separated from the pellet (P1), the supernatant (S1) was then centrifuged again for 15 min at 12 000g. The resulting pellet (P2) corresponds to the crude synaptosome preparation, whereas the supernatant (S2) corresponds to the cleared soluble fraction.

### Purification and Injection of Reelin

#### Reelin Purification

Supernatants containing recombinant full-length Reelin were produced from a stable HEK293 cell line (a gift from Dr Joachim Herz lab, University of Texas Southwestern Medical Center, USA, and Dr Tom Curran, University of Pennsylvania, USA). Conditioned medium from the Reelin-secreting HEK293 cell line or an untransfected HEK293 cell line (control for injection) was collected after 48 h of incubation in serum-free Opti-MEM media. The conditioned media (containing Reelin or lacking Reelin) were concentrated approximately 30-fold using Amicon Ultra 100 000 molecular weight cut off filters (Millipore, Billerica, MA) and dialyzed in PBS according to previously published methods (Forster et al. 2002; Weeber et al. 2002). The purified Reelin content was confirmed by western blotting using the anti-Reelin antibody G10 (Abcam, cat# ab78544) (data not shown). Mass spectrometry analysis revealed >98% of Reelin purity in the Reelin samples with a level of confidence superior to 95% (317 peptides and 1143 spectra for Reelin detected with the Mascot and Scaffold software, respectively) and not a trace of Reelin in the control solution (no peptide, no spectra). Furthermore, there was no toxic molecule (such as cytokines and toxins) in both the Reelin sample and the mock-media control solution. Activity of the purified Reelin was verified by probing the levels of phospho-Serine 473 Akt, a downstream target of the Reelin pathway, in cell lines.

#### Reelin Injection

Mice were anesthetized with 2% isoflurane in 100% O_2_, then positioned with ear bars of a stereotaxic frame. To maintain anesthesia, isoflurane was continuously delivered through a small facemask custom-fitted to the stereotaxic frame. A midline incision was made in the skin overlying the skull. Two holes (AP = –2.3; ML = +/–2.0; DV = 2.0) were drilled through the skull to perform the injection in the dorsal CA1 (Bregma –1.7 to –2.7mm). A solution containing purified Reelin solubilized in PBS (1 μM, 12 μl), Reelin’s central active fragment (R&D systems; Catalog#: 3820-MR-025/CF; 100 μg/mL, 50 μl) or control solution (12 μl) was then injected bilaterally using a Hamilton syringe at a rate of 10 μl/min (Jiang et al. 2016; Kiroski et al. 2020). Immediately after removal of the needle, the skin was closed with sutures.

### Statistics

Omnibus two-factor analysis of variance (ANOVA) was performed on Y-maze acquisition and retention data. Follow ups to one-way ANOVA were performed when the omnibus ANOVAs were significant, and Tukey’s post-hoc *t*-tests were used when the one-way ANOVAs were significant. One-way ANOVA was performed on open field, forced swim, and dry cylinder tasks and when significant they were followed with Tukey's protected *t*-tests. Western-blot data were statistically evaluated using the Student's *t*-test. Tukey’s multiple comparison test was used to compare the area with bedding material in Fig. 1*A*. Data in Fig. 3*C* were compared using the Student’s *t*-test. All figures depict standard deviation.

**Table 1:** Proteins associated with Ndel1 functions and their links to epilepsy and schizophrenia

Protein	Function	Links to epilepsy	Link to schizophrenia	References	Antibodies
Ndel1 (Nuclear distribution protein nudE-like 1)	Regulates the cytoskeleton, contributes to neurodevelopment (see introduction for details)	- ↑ in human epileptic sclerotic hippocampus - Spontaneous recurrent seizures in Ndel1 CKO - ↑ after pilocarpine treatment	- ↑ in first episode of psychosis - ↓ in postmortem brain tissues of schizophrenic patients - Key binding partner of DISC-1	(Ozeki et al. 2003; Lipska et al. 2006; Wu et al. 2014; Choi et al. 2016; Gouvea et al. 2016; Jiang et al. 2016; Gavrilovici et al. 2020; Kiroski et al. 2020)	Home made (rabbit) 1:6000 (Niethammer et al. 2000; Smith et al. 2000; Nguyen et al. 2004)
Nde1 (Nuclear Distribution Factor E homolog 1)	Modulates neurogenesis and cell positioning during cortical development	Nde1 gene mutation is linked to lissencephaly- 4 (LIS4). Microcephaly, cortical lamination deficiency, abnormal MT organization and seizures are typical	- Key binding partner of DISC-1 - ↓ Nde1 impairs process formation in oligodendrocytes and disrupts neuron–oligodendrocyte contact formation and myelination. Oligodendrocytes dysfunction and myelin abnormalities can lead to cognitive deficits and are reported in neuropsychiatric diseases including schizophrenia.	(Feng and Walsh 2004; Burdick et al. 2008; Bradshaw et al. 2009; Alkuraya et al. 2011; Bakircioglu et al. 2011; Shimizu et al. 2018; Schmitt et al. 2019)	Proteintech, 10233-1-AP (rabbit) 1:1000
Lis1 (Lissencephaly 1)	Regulates the molecular motor cytoplasmic Dynein and MTs organization	Lis1 gene mutation is linked to lissencephaly, subcortical band heterotopia (SBH); developmental delay, intellectual disability and epilepsy; embryonic lethality in KO; neuronal migration defects and seldom seizures in heterozygous KO	- Key binding partner of DISC-1 - ↓ Lis1 expression in the brain tissue from patients with schizophrenia - ↓ Lis1 activity results in neuronal migration defects in mammals and early embryonic lethality in rodents	(Reiner et al. 1993; Hirotsune et al. 1998; Sasaki et al. 2000; Guerrini and Carrozzo 2001 ; Leventer et al. 2001; Ross et al. 2001; Lipska et al. 2006; Paredes and Baraban 2002; Jones and Baraban 2007; Wynshaw-Boris 2007; Greenwood et al. 2009; Saillour et al. 2009; Hippenmeyer et al. 2010)	Abcam ab2607 (rabbit) 1:600
GFAP (Glial fibrillary acidic protein)	Modulates the structure and function of astrocytes, most commonly used to detect astrogliosis	↑ in patients presenting epilepsy-associated lesions	↑ in animal models of schizophrenia	(Martinian et al. 2009; Sullivan 2014; Kim et al. 2018)	Sigma, G3893 (mouse) 1:1000
Phospho-Tau (Ser 396 and 404)	Destabilizes MTs when hyper-phosphorylated; Tau normally stabilizes MTs	Hyperphosphorylated in mice with chronic epilepsy and brain samples from drug- resistant chronic TLE patients	Phospho-Tau levels ↓ in schizophrenic patients vs healthy controls	(Demirel et al. 2017; Liu et al. 2018; Alves et al. 2019; Casillas-Espinosa et al. 2020)	Homemade (mouse), a kind gift of Dr. Peter Davies 1:500 (Nguyen et al. 2001; Cruz et al. 2003)
GluR1 (Glutamate receptor 1)	Synaptic plasticity; regulation of postsynaptic membrane potential	Phosphorylation / activation can modulate seizures predisposition and hippocampal hyperexcitability	- KO display features reminiscent of schizophrenia - *GRIA1* is associated to schizophrenia via genome-wide association study	(Wiedholz et al. 2008; Rakhade et al. 2012; Rubio et al. 2012; Amitai and Connors 2013; Barkus et al. 2014)	AB1504 (rabbit) 1:1000
p35	Neuron-specific coactivator of Cdk5; p35/Cdk5 phosphorylates Tau at Se 396/404 (detected with PHF-1 abs)	Cortical lamination defects, seizures, and adult lethality in KO	↓ in postmortem brain tissues of schizophrenia patients	(Chae et al. 1997; Engmann et al. 2011; Shah and Lahiri 2014)	Sc 820(c-19) (rabbit) 1:100
Cdk5 (cyclin-dependent kinase 5)	Phosphorylates Ndel1, regulates the cytoskeleton, contributes to neurodevelopment and neuronal plasticity	↑ seizure susceptibility, ↑ NMDAR-mediated currents and impaired neuronal repolarization in the hippocampus of Cdk5 KO mice	- ↑ in the dorsolateral prefrontal cortex in postmortem schizophrenia brains - ↓ CDK5, p35 and p25 when antipsychotic drugs are used	(Dhavan and Tsai 2001; Sen et al. 2006; Hawasli et al. 2009; Engmann et al. 2011; Su and Tsai 2011; Ramos-Miguel et al. 2013; Lai and Ip 2015; Shupp et al. 2017; Cortés et al. 2019)	Sc-6247(J-3) (mouse) 1:300
CaMKIIα (Calcium/ calmodulin-dependent protein kinase type II subunit alpha)	Regulates synaptic plasticity, neurotransmitter release and long-term potentiation	Modulates Na^+^ current in a mouse model of epilepsy; de novo mutations associated with seizures, neurodevelopmental defects and intellectual disability	Heterozygous KO show features related to schizophrenia	(Novak and Seeman 2010; Küry et al. 2017; Thompson et al. 2017; Akita et al. 2018)	BD, 611292 (mouse) 1:10000
Akt (Protein kinase B)	Pleiotropic kinase involved in multiple cellular processes including neuronal migration and survival	↑ activation of PI3K-Akt-mTORC1 signaling associated with epilepsy, Autism spectrum disorder and intellectual disabilities.	- ↓ p-Akt levels in the dentate gyrus of postmortem tissue from schizophrenic patients - Dysregulation in the anterior cingulate cortex of schizophrenia subjects.	(Wang et al. 2007; Balu et al. 2012; Bradshaw and Porteous 2012; Szamosi et al. 2012; Wu et al. 2013; Itoh et al. 2016; Liu et al. 2017; Manning and Toker 2017; McGuire et al. 2017)	Akt: Cell signaling, #9272 (rabbit) 1:1000 p-Akt: Cell signaling, #4051 (mouse) 1:1500
GSK3β (Glycogen synthase kinase-3 beta)	Involved in energy metabolism and neuronal cell development, downstream target of Akt and phosphorylates Ndel1	- ↑ activity linked to N-methyl-D-aspartate receptor overstimulation and hyperexcitability; - GSK3β alterations ↑ seizure susceptibility in animal models of epilepsy	GSK3 protein levels and/or activity altered in schizophrenic brains	(Lovestone et al. 2007; Hur and Zhou 2010; Weng et al. 2018; Woo et al. 2019; Toral-Rios et al. 2020)	GSK3β Abcam, ab93926 (mouse) 1:1000 p-GSK3β Cell signaling, #9323 (rabbit) 1:1000
PKA (Protein kinase A)	Pleiotropic kinase, regulates Ndel1 functions through DISC-1	- ↑ activation in the rat pilocarpine model of status epilepticus; - ↑ activity linked to KCa3.1 downregulation and ↑ neuronal firing in the epileptic neurons	Activated in the microvascular and perivascular regions of schizophrenic prefrontal cortex	(Bracey et al. 2009; Bradshaw et al. 2011; Nishiura et al. 2017; Tiwari et al. 2019)	Promega, V5340 PepTag nonradioactive kinase assay
DISC-1 (Disrupted in schizophrenia 1 protein)	Neurodevelopment, integration of newly born neurons in the adult brain, neuronal plasticity	KO and epileptic mice exhibit similar granule cell abnormalities	- Gene disrupted in a translocation that segregates with schizophrenia - Truncated mutant-disease protein does not interact with Ndel1	(Ozeki et al. 2003; Bradshaw and Porteous 2012; Wu et al. 2013; Hester and Danzer 2014)	Sc-47990(N-16) (goat) 1:500
TDP-43 (TAR DNA-binding protein 43)	Regulates gene transcription, mRNA splicing, stability and translation	Mesial temporal sclerosis found in TLE is linked to TDP-43 abnormalities	- Associated with frontal dementia – amyotrophic lateral sclerosis - Found in ~70% of sclerotic hippocampus - Mislocalized in patients with late psychosis	(Amador-Ortiz et al. 2007; Velakoulis et al. 2009; Cohen et al. 2011; Aoki et al. 2015)	ProteinTech, 10782-2-Ap (rabbit) 1:2000

MTs: microtubules; TLE: temporal lobe epilepsy; KO: knockout mice; NMDAR: N-methyl-D-aspartate receptor.

## Results

### Ndel1 CKO Mice Display Nest Building Deficits

While maintaining the Ndel1 CKO mice mouse line (*n* = 120), we observed that the bedding material of the mutant mice was dispersed throughout the cage. To determine whether these mice have deficits in nest building, a proxy of well-being and social behavior (Latham and Mason 2004; Jirkof 2014; Rock et al. 2014), we moved Ndel1 CKO mice and their heterozygotes CKO (Het) and WT littermates to new cages with bedding material sparsely distributed. We assessed the distribution of the material 24–72 h later. As shown in Fig. 1*A*, WT and Het mice consistently built tidy nests (Fig. 1*A*). In contrast, Ndel1 CKO mice clearly lacked the ability to perform the task: the bedding material remained randomly scattered throughout the cage (Fig. 1*A*). This phenotype displayed by the Ndel1 CKO was so robust that even blinded, we were able to predict the genotype of the mice based on the performance in this species-specific task.

### Ndel1 CKO Mice are Less Active in Exploratory Dry Surface Tests

When housed in cages, the Ndel1 CKO mice are less active and less prone to explore their environment when compared with their WT and Het littermates (Fig. 1*B*, see Supplementary Videos 1–6: WT, 3–4: Het, and 5–6: CKO). In-cage behavioral ethograms collected during 5 min of video-recordings (Fig. 1*B*) revealed that Ndel1 CKO spent most of their time sitting (~99%). In contrast, WT and Het mice engaged in other behaviors and as such, spent far less time sitting (~50%). Indeed, both groups spent ~25% (~75s) of the time walking and 20–50 s rearing. Both WT mice and Het mice also spent some time obscured by their nest. CKO mice did not have a well-built nest to be obscured by (see Fig. 1*A*). To further characterize the general locomotor activity and exploratory habits of the Ndel1 CKO mice, a cohort of 7 littermates of each genotype was then successively subjected at 7 weeks of age to the Y-maze and open field tasks (Fig. 1*C*,*D*). During the retention phase of the Y-maze task, the 3 groups of mice spent significantly (*F*[2,18] = 3.32, *P* < 0.05) different amounts of time in the novel zone and also traveled significantly (*F*[2,18] = 4.01, *P* = 0.03) different distances. Follow up tests indicated that Ndel1 CKO spent significantly (*P* < 0.05) less time and traveled significantly (*P* < 0.05) less distance in the novel zone when compared with the WT and Het mice indicating a more pronounced avoidance of the novel zone by the mutant mice (Fig. 1*C*). An omnibus 2 by 3 ANOVA revealed that there was no significant main effect of zone (*F*[1, 36] = 1.83, *P* = 0.18) or main effect of genotype (*F*[2,36] = 0.43, *P* = 0.66) or interaction (*F*[2,36] = 1.84, *P* = 0.17) during acquisition of the task indicating no preference for the zones by the mice (Fig. 1*C*). In the open field task, there was a significant (*F*[2,18] = 5.09, *P* = 0.02) main effect of time in the center zone and a significant (*F*[2,18] = 18.26, *P* < 0.0001) main effect of distance in total in the mice (Fig. 1*D*). Ndel1 CKO mice spent significantly (*P* < 0.05) more time in the center zone and traveled significantly (*P* < 0.01) less distance in total than both WT and Het mice (Fig. 1*D*). It is noteworthy that the Ndel1 CKO mice do not have spatio-visuomotor impairment, as demonstrated previously in the Morris water maze task (Kiroski et al. 2020). Taken together, our results indicate that Ndel1 CKO mice are less active in cages and exploratory dry surfaces than their WT and Het littermates.

### Ndel1 CKO Performed Differently in the Forced Swim and Dry Cylinder Tests

The mice were then subjected successively to forced swim and dry cylinder tasks (Fig. 1*E*,*F*). The forced swim task has historically been referred to as a model of depression-like behavior; immobility being a sign of negative mood and despair (Porsolt et al. 1977). However, this interpretation is actively debated as increased immobility is also viewed as a habituation process that would reflect a positive adaptative behavior (Borsini et al. 1986; West 1990; Masuda et al. 2001; de Kloet and Molendijk 2016). In the forced swim test, an ANOVA revealed a significant (*F*[2,18] = 10.20, *P* = 0.001) difference on climbing with Ndel1 CKO mice spending significantly (*P* < 0.01) more time climbing than the other two genotypes (Fig. 1*E*). With respect to time spent floating an ANOVA revealed a significant (*F*[2,18] = 12.06, *P* = 0.0005) difference with Ndel1 CKO mice spending significantly (*P* < 0.01) less time floating compared to the other two genotypes (Fig. 1*F*). In the dry cylinder test that evaluates exploratory tendencies and locomotor asymmetry (Schallert et al. 2000), an ANOVA revealed a significant (*F*[2,18] = 4.43, *P* = 0.03) difference on the time spent touching the glass walls (Fig. 1*F*). Ndel1 CKO mice spent significantly (*P* < 0.05) less time touching the walls (more time not touching the walls) compared with the other two genotypes (Fig. 1*F*). In sum, Ndel1 CKO clearly display atypical behaviors in these 5 tasks when compared with their WT and Het counterparts.

### Molecular Changes in the Hippocampus and Cortex of Ndel1 CKO Mice

Deficit in nest building, reduced exploratory behaviors, and immobility in the forced swim test have been linked to social withdrawal, anxiety, and depression in humans, respectively. The tasks that detect these behavioral differences can be considered to a certain extent as proxies for corresponding human clinical manifestations. The mechanisms that underlie these behaviors have been associated with several molecules linked genetically to human diseases and/or found to be altered in human physiology. In this study, we assessed in both neocortex and hippocampus of Ndel1 CKO mice the levels and/or activity of a dozen proteins linked to Ndel1 functions, and potentially to Ndel1 CKO phenotypes, epilepsy and/or schizophrenia (Nde1, Lis1, GFAP, GluR1, Tau-PHF-1, Cdk5/p35, CaMKIIα, Akt, GSK3β, PKA, DISC-1, and TDP-43; see Table 1 for details).

The CA1 hippocampus of Ndel1 CKO mice undergoes postnatal dispersion (Jiang et al. 2016; Kiroski et al. 2020, Fig. 2*A*). This defect is associated with intense astrogliosis throughout the hippocampus and particularly at the level of the stratum lacunosum moleculare (Fig. 2*A*) that receives inputs from layer III of the entorhinal cortex and contains a specific type of interneuron called the neurogliaform cells. These particular cells mediate feed-forward inhibition of CA1 pyramidal cells and contribute to theta rhythm (Capogna 2011). In contrast, the layering of the neocortex of Ndel1 CKO mice appears grossly unperturbed, as indicated by HE staining (not shown) and Cux1 and Tbr1 staining that marks the layers 2/3 and 6, respectively (Fig. 2*B*, Alcamo et al. 2008). However, there was some astrogliosis in the neocortex (Fig. 2*B*), perhaps indicative of a perturbed hippocampus–neocortex connection. Consistent with the immunofluorescent staining, the levels of GFAP were also found to be upregulated in both neocortex and hippocampus (Fig. 2*C*), as revealed by western-blot analysis. The levels of DISC-1 and TDP-43, 2 molecules related to schizophrenia and amyotrophic lateral sclerosis–frontal temporal dementia (see Table 1) were unchanged in both hippocampal and neocortical tissues of Ndel1 CKO mice when compared with those of WT littermates. The amounts of Cdk5 and CaMKIIα also remained stable in the mutant mice. The levels of Akt, activated (phosphorylated) Akt, GSK3β, inactivated (phosphorylated) GSK3β and the respective p-Akt/Akt and p-GSK3β/GSK3β ratios, indicative of kinase activities, were not different between Ndel1 CKO mice and WT littermates. PKA activity measured with the PepTag nonradioactive kinase assay showed no difference between the two groups in both tissues either. Interestingly, the levels of p35, the neuron-specific coactivator of Cdk5, were slightly but significantly upregulated in the hippocampus (but not in the neocortex) of Ndel1 CKO mice, suggesting that total Cdk5 activity may be higher in that brain region. The p35/Cdk5 complex can phosphorylate the MT-associated protein Tau at serine 396 and 404 (Nguyen et al. 2001; Cruz et al. 2003). Using the PHF-1 antibodies that detected Tau phosphorylated at these residues, we found that the levels of 55 kDa species (the nonfilamentous species) (Jackson et al. 2016) were elevated in the Ndel1 hippocampus when compared with those of WT littermates. This change was not observed in the neocortex, consistent with the much milder phenotypes in that brain tissue. As hyperphosphorylated Tau detaches from MTs and renders them unstable (Ballatore et al. 2007; Morris et al. 2011), our finding of increased PHF-1 signals in the hippocampus of Ndel1 CKO hippocampus are concordant with MT fragmentation found in CA1 pyramidal neurons of these mice (Jiang et al. 2016).

It is noteworthy that the most consistent and striking molecular change that we observed in the Ndel1 CKO is the overexpression of Lis1 (Jiang et al. 2016 and recapitulated in Fig. 2*C*), and this phenotype can be rescued with Reelin (Jiang et al. 2016 and recapitulated in Fig. 3, see below). In our previous studies, we proposed that the Ndel1-Lis1 mis-dosage (i.e., downregulation of Ndel1 and overexpression of Lis1) and related MT fragmentation constitute a central mechanism for CA1 deterioration in Ndel1 CKO mice (Jiang et al. 2016; Kiroski et al. 2020). To follow up on these findings, we next surveyed the levels of Nde1.

*Ndel1* and *Nde1* are paralogues, that is, they originate from the same ancestral genes but occupy different locations in the genome. The two proteins exhibit ~60% identity and ~80% similarity in terms of amino acid sequences. Like Ndel1, Nde1 has also been associated with neurodevelopmental disorders, epilepsy, and schizophrenia (Bradshaw et al. 2013). Interestingly, the two proteins interact with each other and can form hetero-oligomers (Burdick et al. 2008; Bradshaw et al. 2009; Soares et al. 2012). Furthermore, they both bind to DISC-1 Burdick et al. 2008; Bradshaw et al. 2009) and regulate dynein function in a similar way: Ndel1 and Nde1 inhibit dynein motility by dissociating the motor from MTs; however, in association with Lis1, they both stimulate dynein activity (McKenney et al. 2010; Torisawa et al. 2011). For an exhaustive review on the similarities and differences between the expression, regulation and functions of both proteins, please refer to Bradshaw et al. (2013). While Ndel1 is essential for mouse embryogenesis (KO mice are not viable) (Sasaki et al. 2005), Nde1 appears of less necessity: Nde1 KO survive to birth, though with microcephaly (Feng and Walsh 2004). Thus, Ndel1 plays fundamental roles while Nde1 fills in with supporting functions in a cell type/tissue-specific manner (Bradshaw et al. 2013).

Using antibodies specific to Nde1, we found that the levels of Nde1 are downregulated in the hippocampus of Ndel1 CKO. Because Ndel1 and Nde1 can form hetero-oligomers (Burdick et al. 2008; Bradshaw et al. 2009; Soares et al. 2012; Bradshaw et al. 2013), it is possible that the loss of Ndel1 destabilizes Nde1. While Nde1 is important for neurogenesis in the developing brain (Feng and Walsh 2004), its roles in the adult brain and hippocampus remain to be determined. Based on their similar roles in dynein function and MT dynamics (Bradshaw et al. 2013), we reason that Nde1 downregulation (like Ndel1 depletion) contributes to the cytoskeletal disruption in Ndel1 CKO mice.

Because MTs are critical for transport and turnover of receptors and channels, we next determined the levels of a few candidates. We selected GluR1 based on our previous finding in cell cultures showing that Ndel1 affects receptor distribution (Chansard, Hong, et al. 2011a; Chansard, Wang, et al. 2011b). As shown in Figure 2*C*-*E*, the levels of GluR1 decrease significantly in the hippocampus of Ndel1 CKO mice. To define the subcellular localization of GluR1 in Ndel1 CKO hippocampus, we performed crude synaptosome preparations. In WT hippocampus, GluR1 was predominantly enriched in the crude synaptic compartment (P2), with the lowest levels in the soluble fraction (S2) and present in the preclear lysate (S1) and pellet (P1). In the Ndel1 CKO mice (*n* = 6), this distribution was subtly altered with a decrease of 3.2 ± 3.1% (*P* = 0.0468 with *t*-test) in the synaptic fraction when compared with tissues from WT littermates (*n* = 6) (see Fig. 2*F*). Hippocampi of 2 Ndel1 CKO mice out of 6 did not show a decrease of GluR1 in P2 when compared with their WT littermates. This could be explained by the fact that disease progression in the Ndel1 CKO, especially with respect to seizure activity (number of seizures and intensity of seizures per day) is variable (Kiroski et al. 2020), and consequently, not all mice present the same molecular signature with the same intensity at a given age. The potential implication of GluR1 in the phenotypes displayed by the Ndel1 CKO mice is discussed below (see Discussion).

### Reelin Rescues the Nest Building Deficit and Decreases GFAP Upregulation in Ndel1 CKO Mice

In previous studies, we showed that the levels of Reelin glycoprotein are reduced in the hippocampus of the Ndel1 CKO (Jiang et al. 2016) and that supplementation of Reelin via intrahippocampal injection in the mice ameliorates synaptic/dendritic pathologies, reduces intrinsic hyperexcitability of CA1 pyramidal neurons (Jiang et al. 2016), normalizes the levels of Lis1, gene deregulation in the hippocampus and even improves spatial learning and memory function (Kiroski et al. 2020). Furthermore, when treated with Reelin, Ndel1 CKO mice that die from an epileptic phenotype, live twice as long (Kiroski et al. 2020). In this study, we investigate whether Reelin treatment affects the ability of the Ndel1 CKO to build nests. Ndel1 CKO (*n* = 20) were monitored for nest building prior to receiving a single injection of Reelin (full length or the active central fragment). Following the Reelin injection, mice were placed to a new cage with dispersed bedding material. The distribution of the material was assessed prior injection and then after injection over the next 8 days. Within these 8 days, quantification of nesting area was performed with 5 Ndel1 CKO mice injected with Reelin (full-length and central active fragment – see Material and Methods), 3 Ndel1 CKO mice injected with control solution and compared with those from the 11 noninjected mutant mice (Fig. 1*A*), like noninjected mutant mice (Fig. 1*A*), control-injected Ndel1 CKO mice failed to build their nest (Fig. 3*A*,*D*). In contrast, Reelin-injected mutant mice show clear improvement in the task (Fig. 3*B*-*D*). This amelioration conferred with Reelin can be detected as early as 24 h after injection and is sustained for several days.

The proper functioning of astrocytes has been linked to the nest building task. Limiting the nesting material in the first postnatal week of life (from P2 to P9) can trigger early life stress in rodents (Naninck et al. 2015) that in turn, can induce long-term changes in astroglial activity to impact, for example, brain pathology in a mouse model of Alzheimer’s disease (Abbink et al. 2020). Because Ndel1 CKO display higher levels of GFAP in the hippocampus (Fig. 2*A*-*E*) and nesting building deficit exhibited by the mutant mice can be attenuated with Reelin treatment (Figs 1*A* and 3*B*-*D*), we next determined whether Reelin normalizes the levels of GFAP in the Ndel1 CKO hippocampus. We first recapitulated our finding that Lis1 levels are normalized with Reelin treatment (Fig. 3*E*). Importantly, Reelin also attenuates the upregulation of GFAP in the hippocampus of Ndel1 CKO mice to levels found in WT animals (Fig. 3*E*). Taken together, these results indicate that Reelin ameliorates the nest building performance of the Ndel1 CKO and this is associated with reduced glial activation. Of note, Reelin had no effect on the levels of Nde1. Because Ndel1 and Nde1 form hetero-oligomers (Burdick et al. 2008; Bradshaw et al. 2009, 2013; Soares et al. 2012), we reason that the loss Ndel1–Nde1 interaction leads to the destabilization of Nde1, and this defect cannot be overcome with the Reelin treatment.

## Discussion

Epilepsy is typically associated with comorbid conditions including learning and memory deficits, depression, and anxiety. In turn, anxiety is frequently observed in schizophrenia and about 1 patient out of 4 shows depressive behavior. People with schizophrenia also experience cognitive problems, hallucinations, delusions, lack of motivation, and social interest, and in some cases, seizures as a comorbidity. Thus, epilepsy and schizophrenia share several clinical manifestations and/or comorbidities. Genes involved in neurodevelopment and neuronal plasticity in the hippocampus appear to be central in these overlapping diseases (Cascella et al. 2009; Nakahara et al. 2018). In this study, we analyzed the behaviors of Ndel1 CKO mice that exhibit a profound reorganization of the hippocampal CA1 region, spatial learning and memory deficits and ultimately, die prematurely following self-generated recurrent seizures. Dysfunction of Ndel1 is associated with schizophrenia, and the levels of the proteins are also altered in human and models of epilepsy (Lipska et al. 2006; Burdick et al. 2008; Nicodemus et al. 2010; Wu et al. 2014; Choi et al. 2016; Gadelha et al. 2016; Jiang et al. 2016; Bradshaw and Hayashi 2017; Gavrilovici et al. 2020; Kiroski et al. 2020; Zhu et al. 2020). Despite the small cohorts of mice, our behavioral data convincingly show that the Ndel1 CKO mice display clear and robust phenotypic differences from WT and heterozygous CKO littermates (indistinguishable from WT) (Fig. 1). Ndel1 CKO showed reduced species-specific behavior like nest building. They also spent less time in the novel zone on the Y maze test and this may indicate higher levels of fear and anxiety. Ndel1 CKO are generally less active on several dry land tests such as the open field and dry cylinder tests. When forced to swim, they were more active.

Based on the classical interpretation of the forced swim task as a model of depression-like behavior, Ndel1 CKO are unlikely “depressive,” as they appear more active in this task. In support of this interpretation is our finding of higher levels of the astroglial marker GFAP and increased density of GFAP-positive cells in both the hippocampus and neocortex of these mice.

Indeed, decrease (and not increase) in astroglial density has been reported in postmortem prefrontal cortex tissues from patients with major depressive disorder (Rajkowska and Stockmeier 2013; Torres-Platas et al. 2016) and pharmacological depletion of astrocytes in the prefrontal cortex can induce depressive-like behaviors similar to those observed after chronic stress (Banasr and Duman 2008). Alternatively, the higher activity of Ndel1 CKO in the forced swim task could be viewed as a maladaptation to a habituation process (Borsini et al. 1986; West 1990; Masuda et al. 2001; de Kloet and Molendijk 2016) and therefore, to a form of learning. In this regard, the mutant animals display spatial learning and memory deficits as evidenced by the Morris water maze data (Kiroski et al. 2020). It is noteworthy that our Y-maze set-up did not specifically investigate short-term and spatial working memory. Rather, our results indicate a reduced propensity of the Ndel1 CKO mice to explore a novel environment. Combined with the open field results and the reduced exploratory behavior observed in cages, we propose that the Ndel1 CKO may display anxiety-like behaviors.

The abnormal behaviors of Ndel1 CKO likely involve neuronal networks spread throughout several brain regions. The dorsal CA1 hippocampus of Ndel1 is particularly vulnerable to postnatal Ndel1 gene deletion while the anatomy/layering of the neocortex is largely preserved (Fig. 2, Jiang et al. 2016; Kiroski et al. 2020). As the prefrontal cortex and hippocampus are both involved in the innate behavior of nest building (Kolb and Whishaw 1985; Deacon et al. 2002), and the neocortex is apparently unaffected in the Ndel1 CKO mice (Fig. 2), the most straightforward interpretation is that the nesting deficit is due to hippocampal alterations. However, we cannot exclude the possibility that neocortical neurons present subtle alterations that render them impaired. Self-generated recurrent seizures were detected in the Ndel1 CKO using surface electrodes (Kiroski et al. 2020), thereby indicating aberrant neuronal and/or network activity in the neocortex of these mice. The intense astrogliosis in the stratum lacunosum moleculare (Fig. 2*A*) that receives input from layer 3 of the entorhinal cortex (Capogna 2011) further provides evidence of alterations in the cortical tissue of Ndel1 CKO mice, possibly through retrograde transport mechanisms. Thus, a more refined characterization of various brain regions, especially the neocortex, at the morphological, molecular and circuitry levels is required.

Ndel1 CKO mice present self-generated seizures in the hippocampus and neocortex that validate their use to study adult epileptogenesis and for drug discovery (Kiroski et al. 2020). In the Ndel1 CKO hippocampus, there is a reduction of the inhibitory drive from interneurons onto CA1 principal cells, a decrease in the number of symmetric (inhibitory) synapses as well as a decrease in number of calretinin-positive interneurons (Gavrilovici et al. 2020). Together with the intrinsic hyperexcitability displayed by CA1 pyramidal neurons in CKO mice (Jiang et al. 2016), these changes likely explain the hippocampal hyperexcitability in Ndel1 CKO mice and contribute to their seizure activity. Similarly, imbalance in the excitatory/inhibitory inputs caused by NMDA receptor hypofunction (reduced excitatory signaling), and dysfunction and/or loss of interneurons has been advanced for some pathophysiological features and symptoms in schizophrenia (Benes and Berretta 2001; Ross et al. 2006; Alherz et al. 2017; Coyle 2017; Lieberman et al. 2018; Dienel and Lewis 2019; Lee and Zhou 2019). In this context, the hippocampus of Ndel1 CKO mice display a substantial downregulation of genes involved in cell–cell communication, neuronal plasticity, and neurotransmission (Jiang et al. 2016). Here, we found that the levels of the AMPA receptor GluR1 are decreased in the hippocampus of the Ndel1 CKO (Fig. 2) and the distribution of the receptor is subtly altered with a decrease in the synaptic compartment (Fig. 3). This altered trafficking of GluR1 (and perhaps of other receptors and channels) might be caused by Ndel1-Lis1 mis-dosage (i.e., decrease levels of Ndel1 and increased levels of Lis1) that we advanced to be a central mechanism for MT fragmentation and CA1 pathogenesis in Ndel1 CKO mice (Jiang et al. 2016; Kiroski et al. 2020). The decreased levels of the paralogue Nde1 in Ndel1 CKO hippocampi (Figs 2 and 3) may also contribute to disease mechanisms given their similar roles in dynein function and MT dynamics (Bradshaw et al. 2013).

Our data on GluR1 distribution in the Ndel1 CKO mice are compatible with our previous report showing that GluR1 trafficking is altered in cells depleted of Ndel1 and that expression of active Dynamin 2 GTPase partially rescues this defect (Chansard, Hong, et al. 2011a; Chansard, Wang, et al. 2011b). Synaptic GluR1 trafficking in the CA1 region of the hippocampus is required for encoding contextual fear memories (Mitsushima et al. 2011) while alterations in GluR1 have been associated with manic-like behavior (Du et al. 2008). Furthermore, mice lacking GluR1 display schizophrenia-like behaviors (Wiedholz et al. 2008). Thus, the reduced levels of GluR1 in specific neuronal networks might contribute to the intrinsic hyperexcitability and synaptic pathology of CA1 Ndel1 CKO neurons, the memory deficits (Kiroski et al. 2020) and the expression of some of the behavioral deficits described herein for the Ndel1 CKO (Fig. 1). Of note, we did not detect significant differences in the levels and/or activity of CaMKII, Akt, GSK3β, PKA kinases and of molecules associated with diseases such as DISC-1 and TDP-43 in Ndel1 CKO brain tissues. However, we cannot exclude the possibility that these molecules may be altered specifically in the CA1 or in particular subsets of neurons. Therefore, these abnormalities would have not been detected by western blots of whole hippocampal and neocortical lysates.

Importantly, we found a slight but significant increase in levels of the Cdk5 coactivator p35 in the Ndel1 CKO hippocampus (Fig. 2*C*), suggesting an increase in Cdk5 activity in that brain region. The increase in p35 levels was paralleled with an increase in levels of phosphorylated Tau at serines 396 and 404, as indicated by the PHF-1 antibodies. It is noteworthy that Tau is hyperphosphorylated by p25/Cdk5 (for review, see Su and Tsai 2011; Kimura et al. 2014). P25 is a cleavage product of p35 generated by calcium dyshomeostasis and calcium-activated calpain that overactivates Cdk5 (Su and Tsai 2011; Kimura et al. 2014). P25 expression and/or increased p25/p35 ratio contribute(s) to neuronal death through aberrant phosphorylation of multiple cytoplasmic proteins such as Tau and neurofilament proteins (Nguyen et al. 2001; Su and Tsai 2011; Kimura et al. 2014). P25 has been linked to neurofibrillary tangles (Su and Tsai 2011; Kimura et al. 2014), most likely through the hyperphosphorylation of the high molecular species of Tau prone to aggregation. Using antibodies that recognize both p25 and p35 N-terminal, we did not detect p25 in the hippocampus of Ndel1 CKO mice (data not shown). Our finding is consistent with the fact that CA1 Ndel1-depleted hippocampal neurons do not undergo neuronal death (as per neuronal counts with the CA1-specific marker Wsf1—see Gavrilovici et al. 2020), despite the obvious deterioration. Also, only the nonfilamentous Tau is affected in the Ndel1 CKO hippocampus (Fig. 2*C*). Based on these findings, we propose that p35/Cdk5 complex co-operates with other kinases to induce hyperphosphorylation of nonfilamentous Tau in the Ndel1 CKO hippocampus.

In the context of epilepsy, Tau hyperphosphorylation was reported in mice with chronic epilepsy (Alves et al. 2019) as well as in brain samples from drug resistant chronic TLE patients (see review, Casillas-Espinosa et al. 2020). A recent report established that 31 out of 33 epileptic patients (94%) exhibit hyperphosphorylation of the Tau (Fyfe 2016; Tai et al. 2016). Most importantly, the extent of Tau hyperphosphorylation correlated with cognitive dysfunction in these patients (Fyfe 2016; Tai et al. 2016). As hyperphosphorylated Tau detaches from MTs and renders them unstable (Ballatore et al. 2007; Morris et al. 2011), and MTs depolymerization exacerbates the severity and prolongs the duration of SRS in two rat models of adult epilepsy (i.e., pentylenetetrazol [PTZ]-kindling and pilocarpine, these combined results suggest that Tau hyperphosphorylation may contribute to epileptogenesis and spatial learning and memory deficits in the Ndel1 CKO mice; Kiroski et al. 2020).

It is noteworthy that Reelin levels are decreased in patients with schizophrenia (Fatemi 2001; Ishii et al. 2016). Since Reelin signaling can modulate Tau phosphorylation status, it has been proposed that altered transduction of Reelin signaling can lead to Tau hyperphosphorylation and loss of connectivity in schizophrenia (Deutsch et al. 2006). Taken in this context, the reduced levels of Reelin observed in the Ndel1 CKO hippocampus may be related to the increased levels of PHF-1 Tau signals and behavioral deficits. Finally, we found that Reelin improves the nest building ability of the Ndel1 CKO mice and attenuates the upregulation of GFAP in the hippocampus of Ndel1 CKO mice (Fig. 3*E*). These findings add to the striking beneficial effects of Reelin in Ndel1 CKO, at the levels of the CA1 hippocampal function and integrity, spatial learning and memory and lifespan (Jiang et al. 2016; Kiroski et al. 2020) and is in accordance with the involvement of the glycoprotein in the pathophysiology of both epilepsy and schizophrenia (Fatemi 2001; Heinrich et al. 2006; Dazzo et al. 2015; Ishii et al. 2016). Note that the effects of Reelin in other behavioral tests were not tested.

In summary, we propose that the Ndel1 CKO mouse model represents an experimental tool to elucidate the neural mechanisms underlying proxies of clinical phenotypes found in human epilepsy and schizophrenia, and we have provided insights into the molecular signature underlying the pathology and behavioral deficits of these mutant mice. In the future, it would be interesting to extend the behavioral work to assess other aspects of memory, motivation, reward and anxiety, and to compare the effects of Reelin with those of antiepileptic, anxiolytic, and antipsychotic drugs. These future experiments will shed new light on the neuronal networks and signaling pathways that underlie the behavioral deficits in Ndel1 CKO mice and may be instrumental for our understanding of human neuropsychiatric and epileptic conditions.

## Supplementary Material

Videos_1_to_6_tgaa096

Het_first_A_1_mpeg4_tgaa096

Het_first_C_1_mpeg4_tgaa096

Homo_first_D_1_mpeg4_tgaa096

Homo_first_E_1_mpeg4_tgaa096

WT_first_B_1_mpeg4_tgaa096

WT_first_F_1_mpeg4_tgaa096

## Notes

We are grateful to Dr Tom Curran and Dr Joachim Herz for the Reelin-expressing cell line and Dr Mathieu Chansard for insights into this project. *Conflict of Interest*: None declared.
