# Supplementary material for: Behavioral Deficits in Mice with Postnatal Disruption of Ndel1 in Forebrain Excitatory Neurons: Implications for Epilepsy and Neuropsychiatric Disorders
Source: Cereb Cortex Commun. 2021 Jan 7;2(1):tgaa096. doi: 10.1093/texcom/tgaa096 (PMC7876307; doi:10.1093/texcom/tgaa096)

WT 1

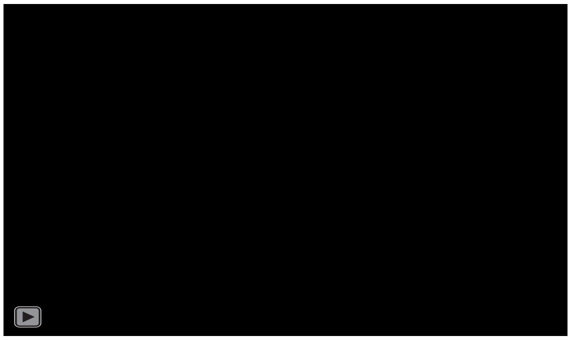

WT 2

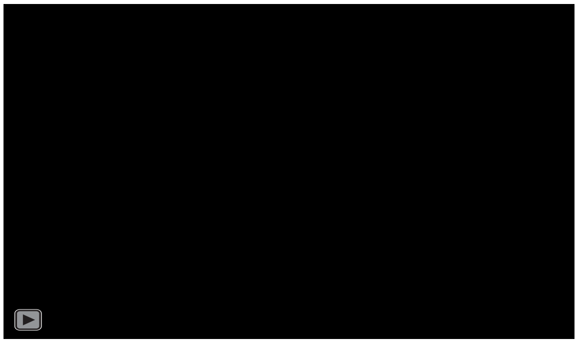

Het 1

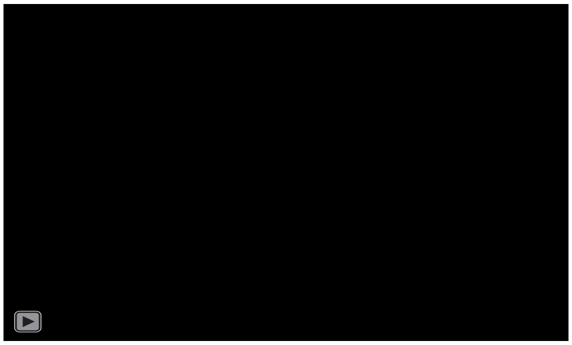

Het 2

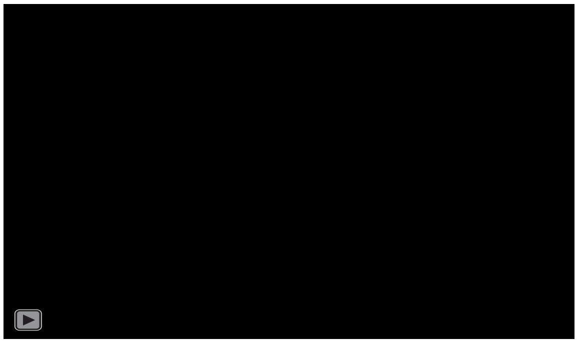

CKO 1

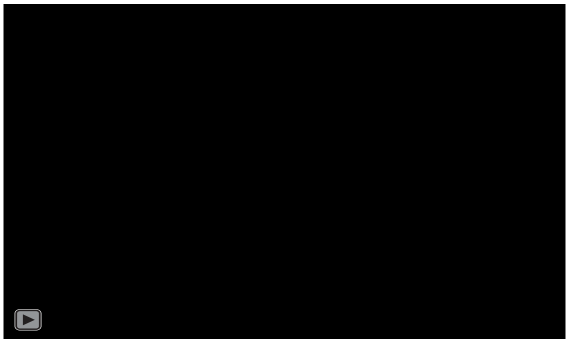

CKO 2

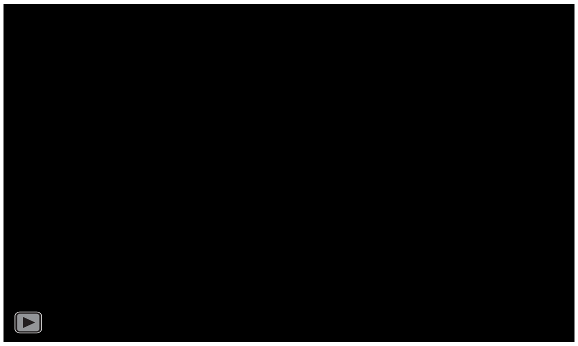

Supplement: Videos_1_to_6_tgaa096 [file videos_1_to_6_tgaa096.pdf]
